# Preventive effect of resistant starch on activated carbon-induced constipation in mice

**DOI:** 10.3892/etm.2013.1096

**Published:** 2013-05-01

**Authors:** YU QIAN, XIN ZHAO, JIANQUAN KAN

**Affiliations:** 1 Department of Food Chemistry and Nutrition, Southwest University, Chongqing 400715;; 2 Department of Biological and Chemical Engineering, Chongqing University of Education, Chongqing 400067, P.R. China

**Keywords:** resistant starch, activated carbon, constipation, bisacodyl, gastrointestinal transit

## Abstract

The aim of this study was to investigate the effects of resistant starch (RS) on activated carbon-induced constipation in ICR mice. ICR mice were fed on diet containing 15% RS of type RS2, RS3 or RS4 for 9 days. Gastrointestinal transit, defecation time and intestinal tissue histopathological sections, as well as motilin (MTL), gastrin (Gas), endothelin (ET), somatostatin (SS), acetylcholinesterase (AChE), substance P (SP) and vasoactive intestinal peptide (VIP) levels in serum were used to evaluate the preventive effects of RS on constipation. Bisacodyl, a laxative drug, was used as a positive control. The time to the first black stool defecation for normal, control, bisacodyl-treated (100 mg/kg, oral administration) and RS2-, RS3- and RS4-treated mice was 78, 208, 109, 181, 144 and 173 min, respectively. Following the consumption of RS2, RS3 and RS4 or the oral administration of bisacodyl (100 mg/kg), the gastrointestinal transit was reduced to 37.7, 52.1, 39.3 and 87.3%, respectively, of the transit in normal mice, respectively. Histopathological sections of intestinal tissue also underscored the protective effect of RS3. The serum levels of MTL, Gas, ET, AChE, SP and VIP were significantly increased and the serum levels of SS were reduced in the mice treated with RS compared with those in the untreated control mice (P<0.05). These results demonstrate that RS has preventive effects on mouse constipation and RS3 demonstrated the best functional activity.

## Introduction

Resistant starch (RS) is defined as the sum of starch and starch-degradation products that are not absorbed in the small intestine due to their resistance to digestive enzymes (1). Based on structure and physicochemical properties, RS may be subdivided into four categories (2): RS1 is a physically inaccessible starch in partially milled grains and seeds; RS2 is a resistant granular starch, such as those in raw potato and banana; RS3 is a retrograded starch, formed in processed foods on cooling (cooled, cooked potato, bread and cornflakes); and RS4 is chemically modified starch. Numerous studies in rats have shown that RS escapes digestion in the small intestine and is slowly fermented in the large intestine to produce short chain fatty acids (SCFAs), lactate and gases (CO_2_,CH_4_ and H_2_) (3–6). RS is reported to have various physiological effects, including weight control, prevention of diabetes, lipid level reduction, promotion of inorganic salt absorption, altering microbial populations and increasing SCFA production in the large intestine (7).

Constipation is defined medically as fewer than three stools per week and severe constipation as less than one stool per week. It occurs when the colon absorbs too much water (8). In the current study, activated carbon was orally administered to mice. Activated carbon attaches to gastrointestinal (GI) mucosal surfaces and reduces the drainage of the GI tract, causes GI fluid reduction and slows down GI movement, resulting in weakness of the spleen and stomach, to produce a model of constipation.

Previous studies have used the constipation model induced by activated carbon to demonstrate the effects of drugs for constipation treatment (9,10). One study demonstrated that a megadose of activated carbon results in digestive tract obstruction (11). Therefore, in the present study, we examined the functional effects of RS in the alimentary tract using an activated carbon-induced constipation mouse model. We examined GI transit, time to the first black stool defecation, histopathological sections and serum assay of motilin (MTL), gastrin (Gas), endothelin (ET), somatostatin (SS), acetylcholinesterase (AChE), substance P (SP) and vasoactive intestinal peptide (VIP) levels. Bisacodyl was used as a positive control. Bisacodyl is a laxative drug that acts as a stimulant of intestinal peristalsis and acts directly on the colon to produce a bowel movement. It is typically prescribed for the relief of constipation and for the management of neurogenic bowel dysfunction, as well as for bowel preparation prior to medical examinations (12–14).

## Materials and methods

### RS preparations

Hylon VII (containing 53% RS2), Novelose 330 (containing 41% RS3) and Novelose 2480 (containing 80% RS4) were supplied by the National Starch and Chemical Co. (Bridgewater, NJ, USA). Casein was obtained from Zhengzhou TianTong Food Ingredients Co., Ltd. (Zhengzhou, China). Soybean oil was purchased from Chongqing Grain Group Co. Ltd. (Chongqing, China). Acetic acid and crotonic acid (standard, purity >99.7%) were obtained from Johnson Matthey (London, UK). Propionic acid, butyric acid and isobutyric acid (standard, purity >99%) were purchased from Tokyo Chemical Industry Co., Ltd. (Shanghai, China). All other chemicals were of reagent grade.

### Animals

Seven-week-old female ICR mice (n=120) were purchased from the Experimental Animal Center of Chongqing Medical University (Chongqing, China). The mice were maintained in a temperature- and humidity-controlled (temperature 25±2°C, relative humidity 50±5%) facility with a 12-h light/dark cycle and free access to a standard rat chow diet and water.

During the experiment, four groups of rats were fed the RS-free basal diet (control group), or a diet containing 15% RS2, 15% RS3 or 15% RS4, respectively. All rats were provided with food and water *ad libitum* and were maintained on each diet for a 4-week period. These experiments followed a protocol approved by the Animal Ethics Committee of Chongqing Medical University (Chongqing, China).

### Induction of constipation in mice

To investigate the preventive effects of RS against activated carbon-induced constipation, the animals were divided into 6 groups with 20 mice in each. The experimental design was as follows: the normal and control groups were fed normal diet for 9 days and the treatment groups were orally fed the RS-free basal diet containing 15% RS2, 15% RS3 or 15% RS4 in their ration, or were fed the RS-free basal diet and treated with a 100 mg/kg dose of bisacodyl dissolved in water. The control and treatment groups received an oral administration of activated carbon (0.2 ml 10% activated carbon, w/w; activated carbon dissolved in 10% arabic gum) at 6 p.m. from the sixth to ninth day to induce constipation.

### GI transit and defecation time

Mice were fasted for sixteen hours from the ninth day at 6 p.m.; however, they were not deprived of water. After 16 h, the mice in the control and treatment groups received an oral administration of 10% activated carbon while the mice in the normal group eceived an oral administration of 10% arabic gum. Thirty minutes later, mice were sacrificed by cervical dislocation under anesthesia with diethyl ether. Ten mice in each group were dissected and the small intestine from the pylorus to the blind intestine was carefully removed. The GI transit of each mouse was calculated as the percentage of the distance traveled by the activated carbon meal relative to the total length of the small intestine. The following equation was used to calculate GI transit: GI transit (%) = distance traveled by the activated carbon/total length of the small intestine ×100. The remaining 10 mice of each group were used to measure the time to the first black stool defecation following the oral administration of 10% activated carbon.

### Histological examination of intestinal tissue

For histological investigations, intestinal tissues were fixed in 10% (v/v) buffered formalin for 24 h, dehydrated in ethanol and embedded in paraffin. Then, 4-*μ*m thick sections were prepared and stained with hematoxylin and eosin (H&E) for observation under an Olympus BX41 microscope (Olympus, Tokyo, Japan).

### MTL, Gas, ET, SS, AChE, SP and VIP levels in serum

MTL, Gas, ET, SS, AChE, SP and VIP levels in the serum were determined using radioimmunoassay kits (Beijing Puer Weiye Biotechnology Co., Ltd., Beijing, China). The serum of mice were collected from heart following surgery.

### Statistical analysis

Data are presented as mean ± standard deviation (SD). Differences between the mean values for individual groups were assessed with one-way analysis of variance (ANOVA) with Duncan’s multiple range test. P<0.05 was considered to indicate a statistically significant difference. SAS version 9.1 (SAS Institute Inc., Cary, NC, USA) was used for statistical analyses.

## Results

### Time to the first black stool defecation

**Figure 1.**
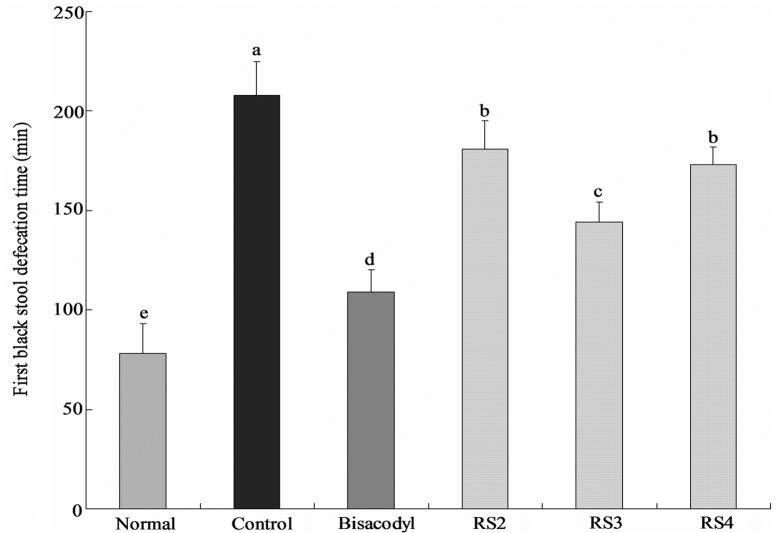
Effects of different types of resistant starch (RS) on the time to first black stool defecation in mice with activated carbon-induced constipation (ICR mice, n=10 per group) compared with 100 mg/kg bisacodyl. ^a–e^Mean values with different letters over the bars are significantly different (P<0.05) according to Duncan’s multiple range test.

The time to the first black stool defecation for each group of mice following the administration of activated carbon, which demonstrates the constipation-inhibiting effect of different treatments, is shown in Fig. 1. The defecation time was the shortest (78±15 min) in the normal group and the longest (208±17 mins) in the control group; the defecation time in the bisacodyl group was 109±11 min, higher than that of the normal group. The times to the first black stool defecation for the RS2, RS3 and RS4 group mice were 181±14, 144±10 and 173±9 min, respectively. According to the defecation time, RS3 has the strongest effect on inhibiting constipation.

### GI transit

**Figure 2.**
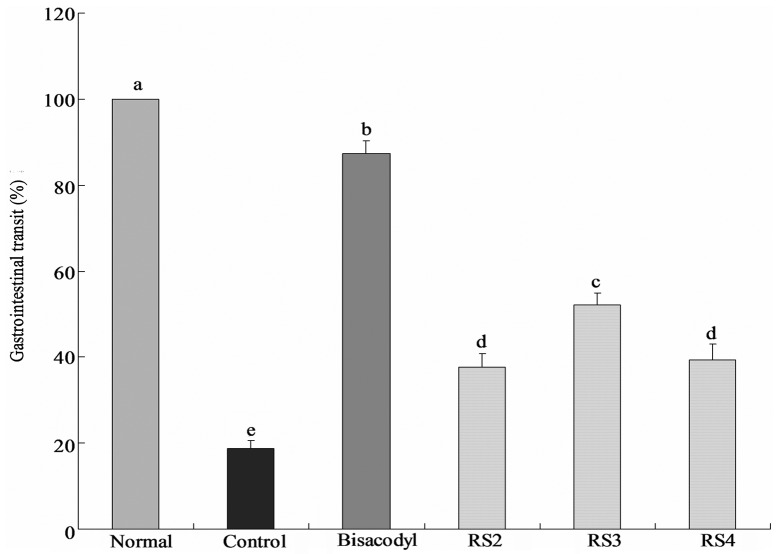
Effects of various types of resistant starch (RS) on gastrointestinal transit in mice with activated carbon-induced constipation (ICR mice, n=10 per group) compared with 100 mg/kg bisacodyl. ^a–e^Mean values with different letters over the bars are significantly different (P<0.05) according to Duncan’s multiple range test.

The constipation inhibiting effects of the treatments were determined by GI transit in mice following the administration of activated carbon (0.2 ml/mouse, 10% activated carbon). In the bisacodyl-treated group, the mean GI transit was 87.3±3.1%, which was higher than that of the control group (18.8±1.8%; Fig. 2). The GI transits of the RS2, RS3 and RS4 groups were 37.7±3.1, 52.1±2.6 and 39.3±3.7%, respectively. RS3 increases the GI transit compared with the control, reduces constipation and increases the functional effect.

### Histopathology of hepatic damage

**Figure 3.**
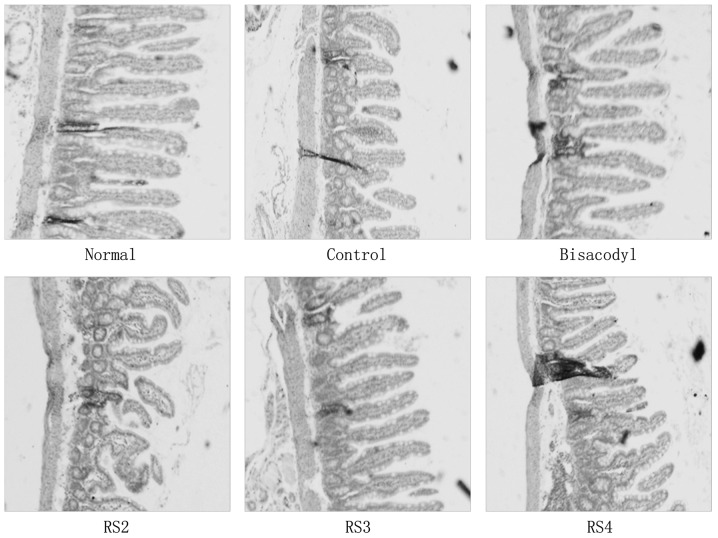
Histological images of intestinal tissues from mice with activated carbon-induced constipation (hematoxylin and eosin staining; magnification, ×40). RS, resistant starch.

H&E staining revealed activated carbon-induced histopathological changes in the intestine; the small intestinal villi became shorter (Fig. 3). The small intestinal villi of the RS3-treated mice were shorter than those of the normal and bisacodyl-treated mice; however, they were longer than those of the RS2- and RS4-treated mice. The small intestinal villi of all RS mice were longer than those of the control mice.

### MTL, Gas, ET, SS, AChE, SP and VIP levels in serum

**Figure 4.**
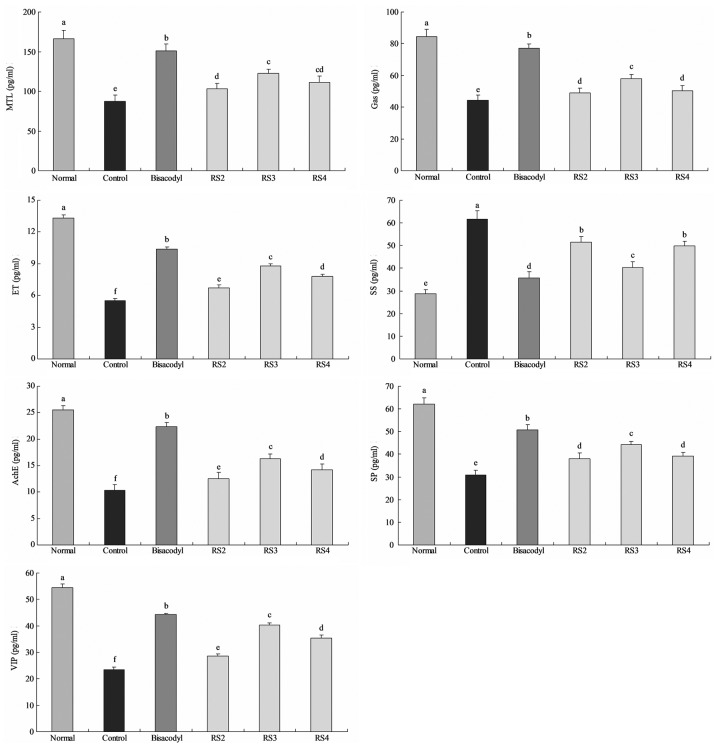
Effect of various types of resistant starch (RS) on serum motilin (MTL), gastrin (Gas), endothelin (ET), somatostatin (SS), acetylcholinesterase (AChE), substance P (SP) and vasoactive intestinal peptide (VIP) levels in mice with activated carbon-induced constipation (ICR mice, n=10 per group) compared with 100 mg/kg bisacodyl. ^a–f^Mean values with different letters over the bars are significantly different (P<0.05) according to Duncan’s multiple range test.

The mean MTL level of normal mice was 166.3±10.2 pg/ml; whereas the MTL level in the activated carbon-induced constipation control mice was reduced to 87.5±7.8 pg/ml (Fig. 4). The level of MTL in mice treated with bisacodyl was 151.3±8.2 pg/ml. The levels of MTL in mice treated with RS2, RS3 and RS4 were 103.4±6.7, 122.2±5.6 and 111.3±7.7 pg/ml, respectively. The Gas levels of the normal, control, 100 mg/kg bisacodyl-, RS2-, RS3- and RS4-treated mice were 84.6±4.4, 44.5±3.1, 77.2±2.6, 48.9±3.1, 57.8±2.7 and 50.2±3.3 pg/ml, respectively. The levels of ET in the RS2, RS3 and RS4 groups were 6.7±0.3, 8.8±0.2 and 7.8±0.2 pg/ml, respectively, and the levels in the normal, control and bisacodyl-treated mice were 13.3±0.3, 5.5±0.2 and 10.4±0.2 pg/ml, respectively. The SS levels of normal and control mice were 28.7±2.0 and 61.6±3.8 pg/ml, respectively, and the SS levels in the treated mice were 35.8±2.6 (100 mg/kg bisacodyl), 51.5±2.5 (RS2), 40.3±2.5 (RS3) and 49.9±2.1 (RS4) pg/ml. The AChE, SP and VIP levels of normal mice were 25.5±0.8, 62.2±2.8 and 54.4±1.4 pg/ml, respectively, and were 22.4±0.7, 50.8±2.2 and 44.3±0.5 pg/ml, respectively, for the bisacodyl-treated mice. The AChE, SP and VIP levels in the RS2 (12.5±1.2, 38.1±2.4 and 28.7±0.8 pg/ml), RS3 (16.3±0.9, 44.2±1.5 and 40.3±0.8 pg/ml) and RS4 (14.2±1.1, 39.2±1.9 and 35.3±1.2 pg/ml)-treated mice were higher than those of the control mice (10.3±1.1, 30.8±2.1 and 23.4±1.1 pg/ml, respectively).

## Discussion

Anorexia is an important symptom in constipation (15). The observation of dietary and water intake in mice may determine the level of constipation and the inhibitory effects of different substances on constipation. The definition of constipation includes infrequent bowel movements and difficulty during defecation (16,17). Constipation most commonly occurs when the stool that forms after food is digested moves too slowly (slow transit) as it passes through the digestive tract. Dehydration, changes in diet and activity, and certain drugs are frequently to blame for the slow transit of stools. When stools move slowly, too much water is absorbed from the stool and it becomes hard and dry (18). Defecation status, dietary intake, water consumption, stool defecation time and GI transit are important standards when investigating constipation.

Histopathology is an important clinical standard in the diagnosis of intestinal function (19). The intestinal villi together increase the intestinal absorptive surface area, providing exceptionally efficient absorption of nutrients in the lumen (20). Intestinal villi also help the intestines to move food along the digestive pathway (21). From our results, we determined that RS3 reduced the damage to intestinal villi in mice treated with activated carbon.

The serum levels of MTL, Gas, ET, AChE, SP and VIP in patients with constipation are lower than those in healthy individuals while the SS levels are higher (22–24). The main function of MTL is to increase the migrating myoelectric complex component of GI motility and stimulate the production of pepsin. It is one of the intestinal hormones responsible for the proper filling and emptying of the GI system in response to intake of food, as well as hunger stimuli and responses (25). Gas is a polypeptide hormone secreted by certain cells of the pyloric glands, which strongly stimulates the secretion of gastric acid and pepsin, and weakly stimulates the secretion of pancreatic enzymes and gallbladder contraction (22). Gas produces effects throughout the GI tract, including promoting GI secretion, increasing GI movement and promoting pyloric sphincter relaxation. ET plays an important role in the stability of vascular tension and maintains the basic cardiovascular system. Constipation not only causes disease, including intestinal obstruction and other serious diseases, but it also induces or aggravates cardiocerebrovascular diseases in the elderly (26). An SS analog, octreotide, has been reported to stimulate intestinal motor complexes and this agent has been used to treat sclerodermatous pseudo-obstruction (27). Stools are formed from the non-digestible components of food after water is either absorbed or secreted in the large intestine. Mucous is also produced in the large intestine to provide viscosity. Thin segments of muscle line the intestinal tract and contract and relax in concert to propel the stool forward. Muscle contraction and mucous secretion are regulated by acetylcholine (28). Patients with slow-transit constipation have abnormal neurotransmitters in the muscular layer of their intestinal walls. These abnormalities include a deficiency of a peptide known as SP, which is thought to contribute to peristalsis (29). Disturbances in the normal neural content of VIP in the bowel wall in idiopathic constipation and diverticular disease may initiate or contribute to the functional changes observed in these disorders (30).

In our previous study (unpublished data), RS3 was demonstrated to produce more SCFAs compared with RS2 and RS4. RS3 demonstrated preventive effects in constipation due to its ability to increase SCFA levels. SCFAs increase the levels of probiotics in the stomach and intestine. SCFA and probiotics have a number of physiological and biological functions, including enhancement of intestinal immunity (31).

The aim of the current study was to investigate whether RS has a preventive effect against activated carbon-induced constipation in mice. In the mice fed with RS3, the results demonstrated that the time to the first black stool defecation was only a little longer than that in mice treated with bisacodyl. The GI transit was longer than that in the control mice and was similar to that in the bisacodyl group. Various serum levels, including MTL, Gas, ET, AChE, SP and VIP in the RS-treated mice were higher compared with those in the control mice and the SS levels demonstrated the opposite tendency. These results suggest that RS has a significant preventive effect on activated carbon-induced constipation in mice and RS3 demonstrated the most potent activity.
